# Understanding Radiology Discrepancies: A Case Cross-Sectional CT Study in a Tertiary Care Setting

**DOI:** 10.7759/cureus.72037

**Published:** 2024-10-21

**Authors:** Vishal Vijayakumar, Krishna Kumar Rama Krishnan, Vigneshwaran S, Prabakaran T, Priyadharshini Bala, Pooja Das

**Affiliations:** 1 Radiodiagnosis, Mahatma Gandhi Medical College and Research Institute, Sri Balaji Vidyapeeth (Deemed to be University), Pondicherry, IND

**Keywords:** brain imaging, clinical significance, cross-sectional study, ct imaging, discrepancy rates, provisional reporting, radiology

## Abstract

Introduction: In modern healthcare, computed tomography (CT) is essential for diagnosing a wide range of medical conditions, particularly in emergency settings where timely evaluation of critical areas such as the brain, thorax, abdomen, and pelvis is crucial. However, the increasing reliance on provisional reports generated by postgraduates during on-call hours introduces challenges, as discrepancies often arise between these initial reports and final assessments by senior radiologists. These discrepancies can affect patient outcomes, particularly in complex cases, underscoring the need for studies that evaluate the patterns and clinical relevance of discrepancies across multiple CT modalities.

Aims and objective: This study aims to evaluate the discrepancy rates between provisional and final radiology reports of cross-sectional CT imaging, focusing on their clinical significance in a tertiary care setting.

Methods: A retrospective analysis was conducted on 1250 CT scans performed during on-call hours at a tertiary care hospital in Pondicherry, India. The study was carried out over one year, from July 2023 to June 2024. It included thoracic, abdominal, pelvic, and brain cross-sectional CT studies. Discrepancies between provisional reports provided by postgraduates and final consultant reports were identified and categorized as major or minor, based on their clinical significance. The American College of Radiology (ACR) RADPEER scoring system was utilized for classification, and statistical analysis was performed to evaluate trends based on postgraduate experience and scan type.

Results: Of the 1250 cases reviewed, 14% exhibited discrepancies between the provisional and final reports, with 6% classified as clinically significant. Abdominal and brain CT scans showed the highest discrepancy rates. A decreasing trend in the rate of discrepancies was observed as postgraduate seniority increased. None of the discrepancies led to adverse clinical outcomes.

Conclusion: The study demonstrates that while provisional reporting by postgraduates is generally accurate, discrepancies, particularly in abdominal and brain imaging, remain a concern. Strengthening training and supervision may help reduce clinically significant discrepancies, thereby enhancing patient safety and care.

## Introduction

In the modern healthcare landscape, where a timely and accurate diagnosis can significantly influence patient outcomes, computed tomography (CT) has established itself as a critical diagnostic tool. CT imaging plays a pivotal role in the evaluation of a wide range of medical conditions, particularly in emergency and trauma settings. Its ability to provide detailed, cross-sectional images of the body enables rapid assessment of critical structures such as the brain, thorax, abdomen, and pelvis. This versatility has made CT a cornerstone in the management of patients with acute presentations, ranging from neurological emergencies to abdominal trauma [1].

However, the increasing demand for CT imaging in emergency settings introduces challenges, particularly when it comes to the accuracy of provisional reports generated by postgraduate residents during on-call hours. These initial reports are often prepared under significant time pressure and with limited resources such as the nonavailability of a senior consultant physically and long working hours. While they provide essential guidance for immediate clinical decision-making, they are subject to revision by senior radiologists [2,3]. The discrepancies between these provisional reports and the final reports, reviewed by experienced consultants, can have important implications for patient care. Such discrepancies, if not identified and addressed in time, may lead to delays in diagnosis, unnecessary additional tests, or even incorrect treatments.

Several factors contribute to these discrepancies, including the level of experience of the reporting postgraduate, the complexity of the clinical case, and the specific body region being examined. Brain and abdominal imaging, for instance, are often more challenging due to the subtlety of certain pathologies and the high stakes involved in misdiagnosing conditions in these regions. Furthermore, the lack of real-time supervision during on-call hours adds to the difficulty, leaving less experienced postgraduates to interpret complex imaging studies on their own.

Previous studies have explored the incidence and causes of discrepancies in radiology reporting, particularly in trauma or system-specific CT scans [4-13]. These studies have highlighted the variability in reporting accuracy based on the body system being examined, with abdominal and thoracic CT scans often showing higher rates of discrepancies compared to other regions. Moreover, the clinical significance of these discrepancies is a subject of ongoing debate, with some studies suggesting that a substantial portion of discrepancies do not lead to adverse clinical outcomes, while others emphasize the need for enhanced training to minimize clinically significant errors.

Despite this body of research, there remains a gap in comprehensive studies that evaluate discrepancy rates across multiple CT modalities, including brain, thoracic, abdominal, and pelvic imaging. Understanding the patterns of discrepancy across these regions is critical for identifying areas where training and supervision may need to be enhanced. Additionally, it is important to assess the clinical relevance of these discrepancies, as not all errors in reporting have equal consequences for patient care.

## Materials and methods

Study design

This study is a retrospective, cross-sectional analysis conducted over one year, from July 2023 to June 2024, in a tertiary care hospital in Pondicherry, India. The study evaluated the discrepancy rates between provisional and final radiology reports for CT scans performed during on-call hours, focusing on thoracic, abdominal, pelvic, and brain cross-sectional imaging. The Institutional Review Board/Independent Ethics Committee (IRB/IEC) approval was waived off as the study was a retrospective analysis, utilizing previously collected data without direct involvement or intervention with participants.

Study participants

A total of 1250 CT scans performed on patients during on-call hours were included in the study. The scans were requested for a variety of clinical indications, including trauma, neurological emergencies, abdominal pain, and thoracic pathologies. Patients of all ages above 18 years and sexes who underwent CT scans of the brain, thorax, abdomen, and pelvis, for whom both provisional reports by postgraduates and final reports by consultants were available, were included in the study. Incomplete imaging studies or reports and patients who underwent non-CT imaging modalities and CT scans with missing provisional or final reports were excluded from the study.

CT imaging protocol

CT scans were performed using a 128-slice CT using (GE OPTIMA). The imaging protocol varied depending on the body region as per the institution's protocol. Brain CT scans were conducted with 120 kVp, 240 mAs, and a slice thickness of 0.625 mm. Non-contrast images were acquired for trauma or stroke patients. Thoracic CT scans were performed using 120 kVp, 200 mAs with or without contrast medium. Axial images were reconstructed at 1.25 mm slice thickness. Abdominal and pelvic CT scans were conducted with 120 kVp, 100 mAs with or without contrast medium. Slice thickness for reconstruction was 1.25 mm. All scans were reviewed on a picture archiving and communication system (PACS). 

Data collection and discrepancy evaluation

Provisional reports were generated by postgraduate junior residents during their on-call duties. The equivalent of a specialty trainee in the UK is referred to as a postgraduate junior resident in India. Final reports were reviewed and issued by consultant radiologists within 24 hours. The senior radiologists who finalized provisional reports are all Doctor of Medicine (MD) Radiodiagnosis degree holders, registered with the Tamil Nadu Medical Council, India. The provisional and finalized reports were compared by senior consultants of Radiology, who did not participate as a duty consultant or finalize that particular case. Discrepancies were defined as differences in interpretation between the two reports that could affect clinical management. These discrepancies were categorized as major and minor discrepancies. Major discrepancies included differences in interpretation that could result in a significant change in diagnosis or treatment. Minor discrepancies included differences that would not alter the patient’s diagnosis or management significantly. The American College of Radiology (ACR) RADPEER scoring system was used to classify discrepancies, as illustrated in Table 1 [14].

**Table 1:** The American College of Radiology (ACR) RADPEER scoring system

Score	Meaning	Optional
1	Concur with interpretation	
2	Discrepancy in interpretation not ordinarily expected to be made (understandable miss)	(a) Unlikely to be clinically significant. (b) Likely to be clinically significant
3	Discrepancy in interpretation/should be made most of the time	(a) Unlikely to be clinically significant. (b) Likely to be clinically significant

Table 2 summarizes the types of undercalled and overcalled discrepancies by RADPEER score category, across inflammation/infection, neoplasia, vascular, and trauma-related findings [15].

**Table 2:** Summary of discrepancies by RADPEER score

RADPEER category	Type of discrepancy	Undercalled discrepancies	Overcalled discrepancies
2a	Understandable miss, unlikely to be clinically significant	-Inflammation/infection: subtle gas from gall bladder fistula, focal lung nodules, mild bronchial thickening. Neoplasia: small pancreatic cyst, thyroid nodule, uterine fibroids	-Inflammation/infection: omental infarction. Neoplasia: gall bladder polyps. Vascular: mild vascular thickening, minor stones (gall bladder/renal)
2b	Understandable miss, likely to be clinically significant	-Inflammation/Infection: pelvic inflammatory disease, perforated appendicitis, renal infection. Neoplasia: subtle liver mass, pancreatic head lesion	-Inflammation/infection: compartmentalized gall bladder abscess, colitis. Vascular: peristalsis misinterpreted as bowel obstruction
3a	Should be detected, unlikely to be clinically significant	-Inflammation/infection: retroperitoneal fluid, pleural effusion. Trauma: known adrenal myelolipoma. Vascular: splenic infarct, myocardial calcification from prior infarct	-Inflammation/infection: centrilobular lung nodularity. Vascular: splenic infarct misinterpreted as splenic injury
3b	Should be detected, likely to be clinically significant	-Inflammation/infection: appendicitis, bowel perforation, tubo-ovarian abscess. Neoplasia: pancreatic head mass, liver metastases, colonic stricture	-Neoplasia: ovarian tumor misinterpreted as fibroid. Vascular: missed portal vein thrombosis, pulmonary embolism. Trauma: missed active retroperitoneal bleed

Statistical analysis

Data were entered and analyzed using IBM SPSS Statistics for Windows, Version 25 (Released 2017; IBM Corp., Armonk, New York, United States). Descriptive statistics were used to summarize demographic data, including age, gender, and type of scan. Categorical variables, including discrepancy types and rates, were expressed as percentages. Chi-square tests were used to evaluate the relationship between the level of postgraduate experience and the rate of discrepancies. A p-value of <0.05 was considered statistically significant.

Patient demographics

Of the 1250 cases included in the study, 54% (675 cases) were male and 46% (575 cases) were female, with ages ranging from 18 to 85 years. The mean age of the patients was 45 ± 15 years. The distribution of male and female patients for each scan type is summarized in Table 3.

**Table 3:** Demographic statistics for each scan type

Scan type	Total cases	Male (%)	Female (%)
Brain CT	450 (36%)	240 (53%)	210 (47%)
Thoracic CT	300 (24%)	165 (55%)	135 (45%)
Abdominal CT	350 (28%)	190 (54%)	160 (46%)
Pelvic CT	150 (12%)	80 (53%)	70 (47%)

## Results

Overall discrepancy rate

**Figure 1:**
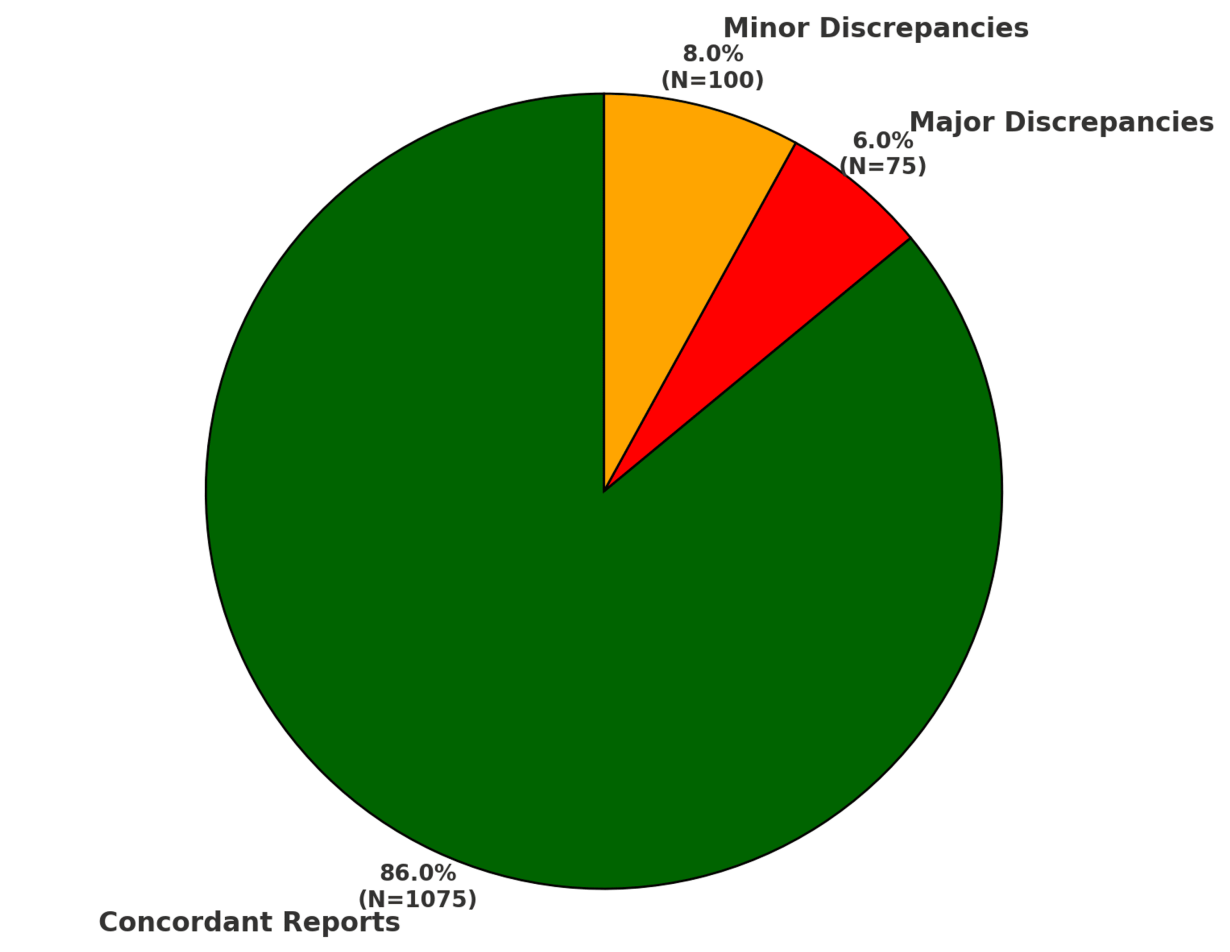
Discrepancy distribution N (total number of reports) = 1250

Of the 1250 CT cases, 175 cases (14%) exhibited discrepancies between the provisional reports generated by postgraduates and the final reports issued by consultants. Among these discrepancies, 75 cases (6%) were classified as major discrepancies (clinically significant), while 100 cases (8%) were minor discrepancies (not clinically significant) as illustrated in Figure 1.

Discrepancies were categorized using the American College of Radiology (ACR) RADPEER scoring system. Table 4 provides a breakdown of the RADPEER scores for the 175 cases with discrepancies.

**Table 4:** Breakdown of discrepancies by RADPEER score

RADPEER score	Definition	Number of cases (%)
1	Concordant interpretation	1075 (86%)
2a	Discrepancy, unlikely to be clinically significant	76 (6%)
2b	Discrepancy, likely to be clinically significant	34 (3%)
3a	Discrepancy, should be detected, not clinically significant	24 (2%)
3b	Discrepancy, should be detected, clinically significant	41 (3%)

**Figure 2:**
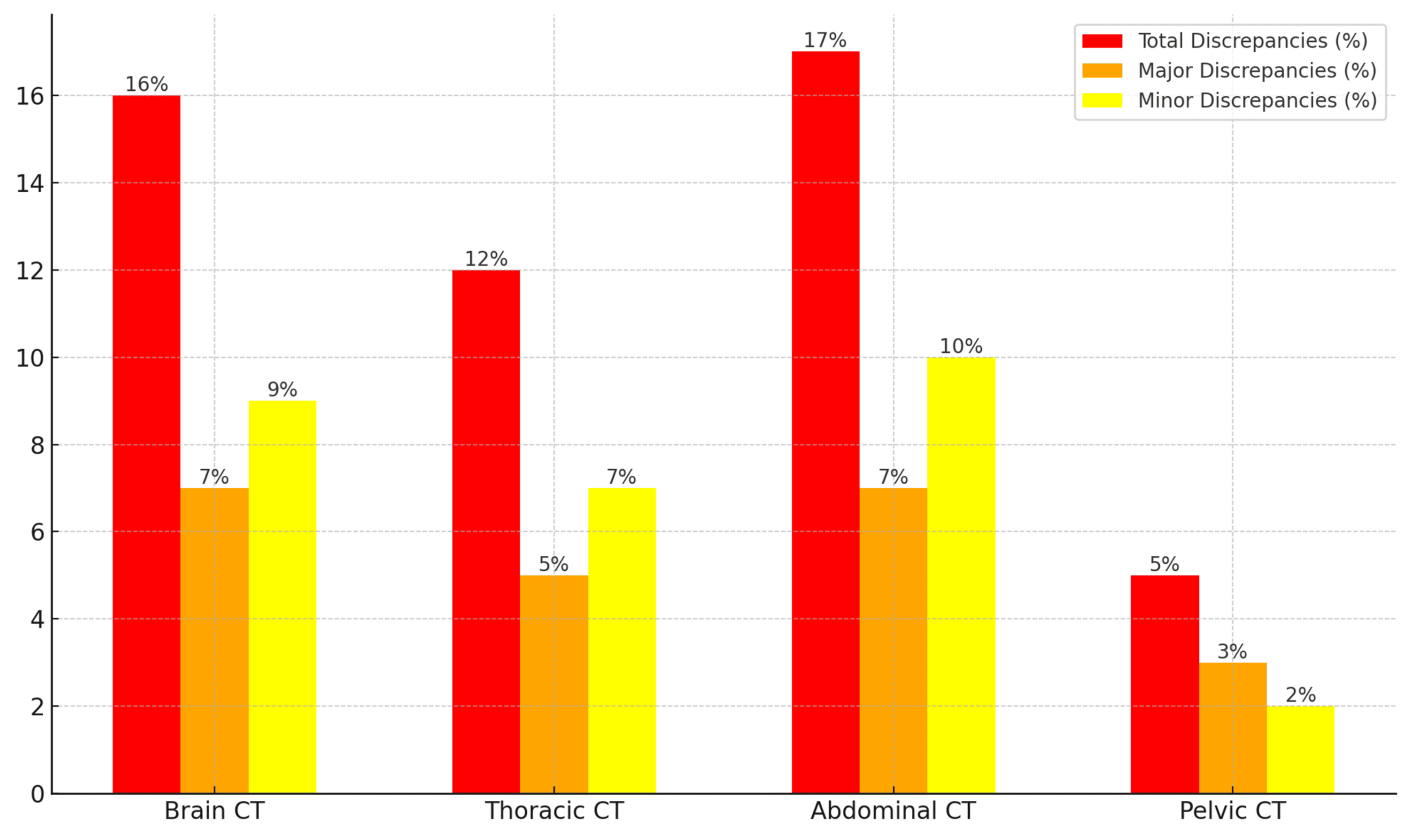
Discrepancy distribution by scan type

The distribution of discrepancies varied across different types of CT scans. Brain and abdominal CT scans had the highest number of discrepancies, accounting for 60% of the total discrepancies. Table 5 and Figure 2 provide a summary of the discrepancy rates by scan type.

**Table 5:** Discrepancy rates by CT scan type

Scan type	Total cases	Total discrepancies (%)	Major discrepancies (%)	Minor discrepancies (%)
Brain CT	450	72 (16%)	30 (7%)	42 (9%)
Thoracic CT	300	35 (12%)	15 (5%)	20 (7%)
Abdominal CT	350	60 (17%)	25 (7%)	35 (10%)
Pelvic CT	150	8 (5%)	5 (3%)	3 (2%)

Undervalued and Overcalled Discrepancies

Of the 175 total discrepancies, 110 cases (63%) involved undervalued findings, where the provisional report missed or underreported a significant finding. Additionally, 65 cases (37%) were classified as overcalls, where the provisional report described findings that were not confirmed by the final report.

Discrepancies by Postgraduate Seniority

A decreasing trend in the rate of discrepancies was observed as postgraduate seniority increased. Table 6 shows the breakdown of discrepancy rates across the three postgraduate years.

**Table 6:** Discrepancy rate by postgraduate seniority Note: p-values are calculated using the Chi-square test comparing each year with Year 1. Additionally, the decreasing trend in discrepancy rates with increasing postgraduate seniority was statistically significant (p < 0.001) using the Cochran-Armitage test for trend, indicating that greater experience resulted in more accurate provisional reports

Postgraduate year	Total cases	Total discrepancies (%)	p-value	Chi-square values
Year 1	400	72 (18%)	Reference	-
Year 2	450	50 (11%)	0.004	7.62
Year 3	400	30 (7%)	<0.001	18.89

**Figure 3:**
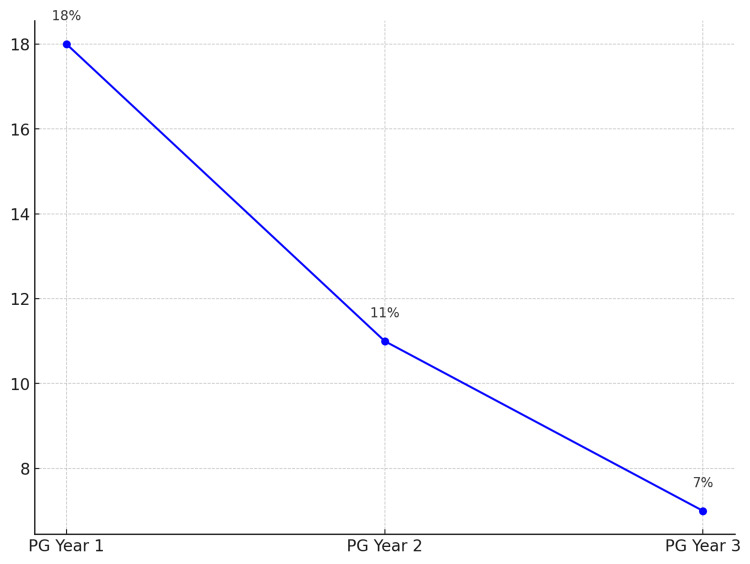
Discrepancy rate among postgraduate PG: Postgraduate

The highest discrepancy rate was seen in Year 1 postgraduates, with 18% of cases showing discrepancies, while Year 3 postgraduates had the lowest rate at 7%, as illustrated in Figure 3. This decrease in discrepancy rates with increasing postgraduate seniority was statistically significant (p < 0.05), indicating that greater experience resulted in more accurate provisional reports.

Clinical impact of major discrepancies

Among the 75 major discrepancies, only 10 cases resulted in delayed treatment, but none led to adverse clinical outcomes. In all cases where major discrepancies were identified, corrective actions were taken based on the final reports, ensuring appropriate management.

## Discussion

In this retrospective analysis of 1250 cross-sectional CT scans, we evaluated discrepancies between provisional reports prepared by postgraduates and final reports issued by consultant radiologists. Our study's overall discrepancy rate of 14% is in line with previous research, such as that by Phua et al, which reported a 15.1% discrepancy rate in cross-sectional imaging. In their study, major discrepancies were noted in 7.3% of cases, comparable to our findings of 6% [4]. This suggests that while provisional reporting by postgraduates is generally accurate, there remain clinically significant errors that could alter patient management if not corrected.

Brain and abdominal CT scans exhibited the highest rates of discrepancies, with abdominal CT showing 17% and brain CT showing 16%. This trend has been observed in previous studies, as the interpretation of abdominal and brain imaging can be particularly challenging due to subtle findings and complex anatomy. For instance, Briggs et al. reported that subtle hemorrhages or small soft tissue masses in the brain are frequently missed in preliminary reports [16].

The RADPEER scoring system was used to classify discrepancies in our study [14]. Most of the discrepancies were graded as RADPEER 2a (minor discrepancies unlikely to affect clinical outcomes), while 3% of cases were categorized as RADPEER 3b (clinically significant and should have been identified). This breakdown is consistent with reports by Ruchman et al., who also found a higher prevalence of minor discrepancies compared to major ones [17]. The classification of discrepancies according to RADPEER scores provides a structured way to assess and address reporting errors, focusing on reducing clinically significant discrepancies.

**Figure 4:**
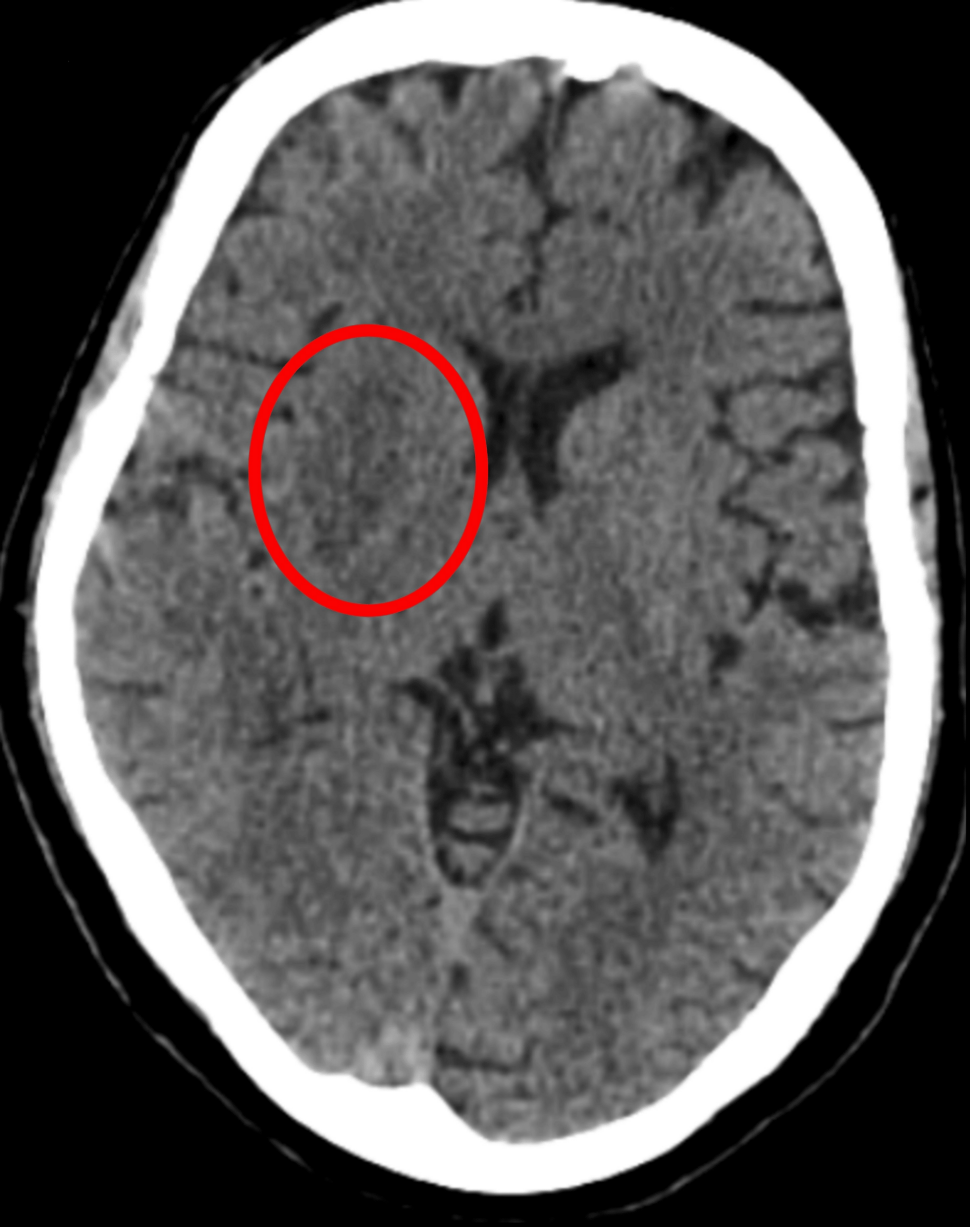
CT brain of a 58 years old, who presented with left side weakness The image shows a hypodense area involving the right capsuloganglionic region, suggestive of acute nonhemorrhagic infarct, which was missed in the provisional report. Original CT brain image of a patient clinically suspected of acute infarct

In our study, 63% of the discrepancies involved undervalued findings, cases where significant findings were either missed or underreported. This is similar to reports by Phua et al. and Carney et al., who found that subtle findings, particularly in brain and abdominal CT, were often missed in provisional reports [4,11]. Figure 4 demonstrates an undercalled finding in brain CT. The remaining 37% of discrepancies were overcalled findings, where normal structures or benign conditions were mistakenly flagged as pathological. For example, in several cases, calcifications in the abdomen were incorrectly reported as suspicious for malignancy.

Overcalls are particularly problematic because they can lead to unnecessary additional testing or interventions. However, they are generally less harmful than undervalued findings, where a missed diagnosis can delay necessary treatment. Briggs et al. noted similar findings in their study on polytrauma CT, where overcalled findings included normal anatomical variations being mistaken for fractures or other pathologies [6,7].

A significant finding of our study was the inverse relationship between postgraduate seniority and discrepancy rates. Year 1 postgraduates had the highest discrepancy rate at 18%, while Year 3 postgraduates had the lowest at 7%. This trend suggests that as postgraduates gain experience, their diagnostic accuracy improves, a finding consistent with studies conducted by Hillier et al. and Tieng et al., who also reported decreasing error rates with increasing experience [9,18].

When compared to studies like Briggs et al. (2010), our overall discrepancy rate of 14% is lower than the 24% discrepancy rate observed in polytrauma CT cases . This difference may be due to the broader range of CT studies included in our research, as opposed to the more complex trauma cases assessed in polytrauma studies. Nonetheless, the major discrepancy rate (6%) in our study is comparable to that found in polytrauma CT, reinforcing the importance of consultant review, especially in high-stakes imaging.

Recommendations and suggestions to improve CT reporting accuracy by on-call residents and to reduce errors should be formulated at every institutional level. A peer review study from Cincinnati Children’s Hospital​ concluded that discrepancies in interpretations should trigger a peer review process. Conducting bimonthly peer review conferences where errors and challenging cases are openly discussed encourages learning from mistakes. Regular interdisciplinary case reviews and discussions on discrepancies help reinforce learning from missed or incorrectly interpreted cases [19].

Tools like Orion​, which monitor discrepancies in resident and fellow reporting, can be adapted for overall radiology departments. Automatic detection of discrepancies between preliminary and final CT reports can help ensure that every significant discrepancy is caught and analyzed. Residents and radiologists should receive timely feedback on discrepancies​ [20].

The double-reading protocol highlights the value of a second review of CT scans by experienced radiologists within 12 hours of the initial interpretation. The study suggests that errors during the first interpretation are often due to the urgency and stress of trauma settings, as well as reduced attention during night shifts​. Implementing dedicated protocols for night shifts, such as mandatory double reading the following day, can help mitigate the effects of fatigue on diagnostic accuracy [21].

Study limitations

As with any retrospective study, there are limitations to consider. First, we relied on the final consultant report as the "ground truth," rather than conducting a blinded re-review of the images. This may have introduced some bias into our assessment of discrepancies. Secondly, our study focused on CT cross-sectional imaging of specific body regions (brain, thorax, abdomen, and pelvis) and did not include subspecialty imaging such as musculoskeletal or vascular CT, which may have different discrepancy rates. Future studies could benefit from including a wider range of imaging modalities .

## Conclusions

Our findings highlight the importance of structured reporting systems, postgraduate training, and consultant oversight in reducing clinically significant discrepancies. While provisional reporting is generally accurate, discrepancies remain, particularly in complex imaging like brain and abdominal CT. Implementing structured feedback mechanisms and providing additional training in these high-risk areas could further improve diagnostic accuracy. Moreover, future research should explore the role of artificial intelligence in supporting radiologists during on-call hours, particularly for identifying subtle findings that are commonly missed.

## References

[1] Rosen MP, Sands DZ, Longmaid HE 3rd, Reynolds KF, Wagner M, Raptopoulos V (2000). Impact of abdominal CT on the management of patients presenting to the emergency department with acute abdominal pain. AJR Am J Roentgenol.

[2] Shah NA, Hoch M, Willis A, Betts B, Patel HK, Hershey BL (2010). Correlation among on-call resident study volume, discrepancy rate, and turnaround time. Acad Radiol.

[3] Davenport MS, Ellis JH, Khalatbari SH, Myles JD, Klein KA (2010). Effect of work hours, caseload, shift type, and experience on resident call performance. Acad Radiol.

[4] Kia-Sheng Phua J, Tim-Ee Cheng L (2022). Evaluating discrepancy rates of radiology resident provisional reports for cross-sectional body imaging studies at a tertiary hospital. Proc Singap Healthc.

[5] Miyakoshi A, Nguyen QT, Cohen WA, Talner LB, Anzai Y (2009). Accuracy of preliminary interpretation of neurologic CT examinations by on-call radiology residents and assessment of patient outcomes at a level I trauma center. J Am Coll Radiol.

[6] Wu Y, Das B, Shah V, Verma R, Stephenson JA (2020). An audit of local discrepancy rates in acute abdominal CT: does subspecialist reporting reduce discrepancy rates?. Clin Radiol.

[7] Ruma J, Klein KA, Chong S, Wesolowski J, Kazerooni EA, Ellis JH, Myles JD (2011). Cross-sectional examination interpretation discrepancies between on-call diagnostic radiology residents and subspecialty faculty radiologists: analysis by imaging modality and subspecialty. J Am Coll Radiol.

[8] Stevens KJ, Griffiths KL, Rosenberg J, Mahadevan S, Zatz LM, Leung AN (2008). Discordance rates between preliminary and final radiology reports on cross-sectional imaging studies at a level 1 trauma center. Acad Radiol.

[9] Tieng N, Grinberg D, Li SF (2007). Discrepancies in interpretation of ED body computed tomographic scans by radiology residents. Am J Emerg Med.

[10] Ruutiainen AT, Scanlon MH, Itri JN (2011). Identifying benchmarks for discrepancy rates in preliminary interpretations provided by radiology trainees at an academic institution. J Am Coll Radiol.

[11] Carney E, Kempf J, DeCarvalho V, Yudd A, Nosher J (2003). Preliminary interpretations of after-hours CT and sonography by radiology residents versus final interpretations by body imaging radiologists at a level 1 trauma center. AJR Am J Roentgenol.

[12] Cooper VF, Goodhartz LA, Nemcek AA Jr, Ryu RK (2008). Radiology resident interpretations of on-call imaging studies: the incidence of major discrepancies. Acad Radiol.

[13] Siegle RL, Baram EM, Reuter SR (1998). Rates of disagreement in imaging interpretation in a group of community hospitals. Acad Radiol.

[14] Goldberg-Stein S, Frigini LA, Long S, Metwalli Z, Nguyen XV, Parker M, Abujudeh H (2017). ACR RADPEER Committee White Paper with 2016 updates: revised scoring system, new classifications, self-review, and subspecialized reports. J Am Coll Radiol.

[15] Maloney E, Lomasney LM, Schomer L (2012). Application of the RADPEER™ scoring language to interpretation discrepancies between diagnostic radiology residents and faculty radiologists. J Am Coll Radiol.

[16] Briggs RH, Rowbotham E, Johnstone AL, Chalmers AG (2010). Provisional reporting of polytrauma CT by on-call radiology registrars. Is it safe?. Clin Radiol.

[17] Ruchman RB, Jaeger J, Wiggins EF 3rd, Seinfeld S, Thakral V, Bolla S, Wallach S (2007). Preliminary radiology resident interpretations versus final attending radiologist interpretations and the impact on patient care in a community hospital. AJR Am J Roentgenol.

[18] Hillier JC, Tattersall DJ, Gleeson FV (2004). Trainee reporting of computed tomography examinations: do they make mistakes and does it matter?. Clin Radiol.

[19] Itri JN, Kim W, Scanlon MH (2011). Orion: a web-based application designed to monitor resident and fellow performance on-call. J Digit Imaging.

[20] Halsted MJ (2004). Radiology peer review as an opportunity to reduce errors and improve patient care. J Am Coll Radiol.

[21] Agostini C, Durieux M, Milot L, Kamaoui I, Floccard B, Allaouchiche B, Pilleul F (2008). Value of double reading of whole body CT in polytrauma patients (Article in French). J Radiol.

